# A Mitochondrial Kinase Complex Is Essential to Mediate an ERK1/2-Dependent Phosphorylation of a Key Regulatory Protein in Steroid Biosynthesis

**DOI:** 10.1371/journal.pone.0001443

**Published:** 2008-01-16

**Authors:** Cecilia Poderoso, Daniela P. Converso, Paula Maloberti, Alejandra Duarte, Isabel Neuman, Soledad Galli, Fabiana Cornejo Maciel, Cristina Paz, María C. Carreras, Juan J. Poderoso, Ernesto J. Podestá

**Affiliations:** 1 Instituto de Investigaciones Moleculares de Enfermedades Hormonales, Neurodegenerativas y Oncológicas (IIMHNO), Department of Human Biochemistry, School of Medicine, University of Buenos Aires, Buenos Aires, Argentina; 2 Laboratory of Oxygen Metabolism, University Hospital, University of Buenos Aires, Buenos Aires, Argentina; 3 Department of Clinical Biochemistry, University Hospital, University of Buenos Aires, Buenos Aires, Argentina; University of Washington, United States of America

## Abstract

ERK1/2 is known to be involved in hormone-stimulated steroid synthesis, but its exact roles and the underlying mechanisms remain elusive. Both ERK1/2 phosphorylation and steroidogenesis may be triggered by cAMP/cAMP-dependent protein kinase (PKA)-dependent and-independent mechanisms; however, ERK1/2 activation by cAMP results in a maximal steroidogenic rate, whereas canonical activation by epidermal growth factor (EGF) does not. We demonstrate herein by Western blot analysis and confocal studies that temporal mitochondrial ERK1/2 activation is obligatory for PKA-mediated steroidogenesis in the Leydig-transformed MA-10 cell line. PKA activity leads to the phosphorylation of a constitutive mitochondrial MEK1/2 pool with a lower effect in cytosolic MEKs, while EGF allows predominant cytosolic MEK activation and nuclear pERK1/2 localization. These results would explain why PKA favors a more durable ERK1/2 activation in mitochondria than does EGF. By means of *ex vivo* experiments, we showed that mitochondrial maximal steroidogenesis occurred as a result of the mutual action of steroidogenic acute regulatory (StAR) protein –a key regulatory component in steroid biosynthesis-, active ERK1/2 and PKA. Our results indicate that there is an interaction between mitochondrial StAR and ERK1/2, involving a D domain with sequential basic-hydrophobic motifs similar to ERK substrates. As a result of this binding and only in the presence of cholesterol, ERK1/2 phosphorylates StAR at Ser^232^. Directed mutagenesis of Ser^232^ to a non-phosphorylable amino acid such as Ala (StAR S232A) inhibited *in vitro* StAR phosphorylation by active ERK1/2. Transient transfection of MA-10 cells with StAR S232A markedly reduced the yield of progesterone production. In summary, here we show that StAR is a novel substrate of ERK1/2, and that mitochondrial ERK1/2 is part of a multimeric protein kinase complex that regulates cholesterol transport. The role of MAPKs in mitochondrial function is underlined.

## Introduction

In the complex process of steroidogenesis, the mitochondria is the site where the rate limiting step -cholesterol transport across the mitochondrial membranes- occurs [1], [2].

Cholesterol transport requires specific interactions at the mitochondria between several proteins including the voltage-dependent anion channel (VDAC) [3], the peripheral benzodiazepine receptor (PBR, currently named translocator protein or TSPO) [4], the PBR-associated protein (PAP7) [5], and the steroidogenic acute regulatory protein (StAR) [5]–[8]. The StAR protein, which has the mitochondrial target sequence at the N terminus, is synthesized as a 37 kDa precursor protein in the cytosol, which is cleaved in the mitochondrial matrix to form a 30 kDa protein [7], [9]–[11]. The N terminal 47 or 62 aminoacid-truncated murine or human forms of StAR protein stimulates cholesterol transport outside the mitochondria, indicating that the 30 kDa form is active at the outer mitochondrial membrane [12], [13]. According to the results described above, the active form of StAR should be re-exported after the processing of the precursor in the matrix. This hypothesis is not proved yet. Nevertheless, it is known that the re-export process exists for other proteins such as HSP60 [14]. Additionally, it has also been shown that the inhibition of the processing of the 37 kDa form of StAR protein inhibits steroidogenesis in MA-10 Leydig and Y1 adrenocortical cells [9], [11], [15]–[18]. Thus, the relation between the processing efficiency of StAR protein in mitochondria and the steroidogenic activity in cells of the active form of StAR has not been fully clarified.

The transcription of the StAR gene increases in a cAMP-dependent protein kinase (PKA)-dependent manner [6], [19]. In addition, the non-genomic post-translational effects of PKA have been reported in relationship to StAR. PKA phosphorylates murine and human StAR at specific motifs like Ser^56-57^ and Ser^194-195^, respectively [20]. This event is required for StAR function [21]. Although it is certain that PKA activation is important for trophic hormone-stimulated steroid biosynthesis [22]–[26], it is also known that ERK1/2 and its upstream activator MEK1/2 participate in the regulation of steroidogenesis by genomic and non-genomic effects [27]–[32]. However, little is known about their role in the non-genomic effects. Several groups, including ours, have shown that ERK1/2 and MEK1/2 are targeted to mitochondria in different tissues [33], [34]. Given that mitochondria are the main location for acute regulation of steroidogenesis, the aim of this study was to evaluate the role of ERK1/2 in the mitochondria of steroidogenic cells. Herein we demonstrated a PKA-dependent activation of mitochondrial ERK1/2, which in turn interacts with and phosphorylates StAR in a cholesterol-dependent fashion.

In summary, we have demonstrated that mitochondrial ERK1/2 is a regulator of cholesterol transport, emphasizing that the subcellular localization of MAPKs is an important mechanism in the regulation of specific cell functions.

## Results

### MEK1/2 activation and active ERK1/2 are strictly required for steroidogenesis and are dependent on PKA activation

**Figure 1:**
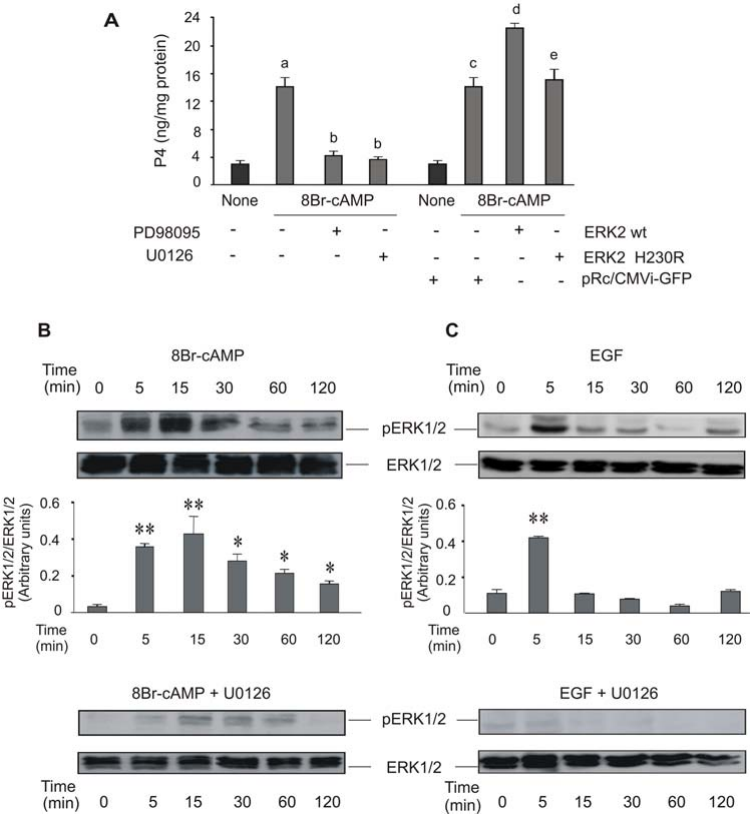
MEK1/2 activation and active ERK1/2 are strictly required for steroidogenesis. (A) MA-10 cells were pre-incubated for 30 minutes with 50 µM of PD98059 or 10 µM of U0126. Then, they were treated with or without 0.5 mM of 8Br-cAMP for 15 minutes. Progesterone (P4) was measured by radioimmunoassay (RIA) as previously described [41], [60] (a, p<0.05 vs. control; b, p<0.05 vs. 8Br-cAMP alone). MA-10 cells were also transfected by electroporation using two plasmids, bearing the sequence of wild-type or a mutated form of ERK2 (H230R). Data are expressed as means±SD of three independent experiments. c, p<0.05 vs. control transfection pRc/CMVi-GFP; d, p<0.05 vs. transfected cells+8Br-cAMP; e, p<0.05 vs. wild-type ERK2). After the incubation with or without 10 µM of U0126, MA-10 cells were stimulated with 0.5 mM of 8Br-cAMP (B) or 10 ng/ml of EGF (C) for the indicated times. Cell lysates were subjected to SDS-PAGE and Western blot as previously described [41], with specific antibodies against pERK1/2 and total ERK1/2 sequentially. The immunoblots show a representative result of three independent experiments. The intensity of the bands was quantitated using total ERK1/2 as loading control. Bars denote relative levels of pERK1/2 presence in arbitrary units. Data are expressed as means±SD of three independent experiments. **, p<0.01 vs. 0 min.

Fig. 1A shows that treatment with U0126 or PD98095 (inhibitors of the activation of MEK1/2) inhibited production of progesterone (P4) by MA-10 Leydig cells stimulated with 8Br-cAMP during 15 minutes. These findings confirm those by Gyles *et al*. and Manna and Stocco [27], [29]. However, the results do not agree with those by Seger *et al*. which show that ERK signaling cascade inhibited gonadotropin-stimulated steroidogenesis [31]. It is worth to mention that in this paper, there are several methodological differences, being the main of them that the effect of MEK/ERK inhibitors are tested after 24 and 48 hs of stimulation of rLHR4 granulosa cells with gonadotropin [31].

The effects we observed with U0126 and PD98095 were not mediated by inhibition of PKA, since this enzyme remained fully active in the presence of both inhibitors (data not shown). Again in line with previously published results [30], [35], [36], U0126 and PD98095 had no effects on the cytochrome P450 cholesterol side chain cleavage enzyme because 22(R)-OH-cholesterol, a freely diffusible analogue of cholesterol, initiated steroid production even when the inhibitors were in the culture media (data not shown).

Active ERK1/2 was confirmed to be necessary for steroidogenesis. The overexpression of a wild-type form of ERK2 in MA-10 cells produced an increase in steroid production stimulated by submaximal concentrations of 8Br-cAMP (Fig. 1A). Overexpression of wild-type ERK2 resulted in an increase of about 45% in steroid production and comprised about 70% of maximal steroidogenesis (data not shown). The 45% increase is explained by the abundance of active ERK2 in the transfected cells. It is worth to mention here that the cell transfection efficiency in these experiments is about 35–40%, as determined by green fluorescence protein transfection control, and it is in accordance with the bibliography [37], [38]. An inactive form of ERK2, the H230R variant, which fails to interact with MEK1, but retains the ability to interact with MEK2 in a weakened fashion [39] did not produce the effect of wild-type ERK2 (Fig. 1A).

Next, we exposed MA-10 cells to differential stimulation with epidermal growth factor (EGF) or with cAMP. Physiologically, whereas cAMP analogues such as 8Br-cAMP maximally stimulate steroid production in Leydig cells, EGF only produces 10–20% of the maximal steroid production compared to cAMP [40]. Regarding ERK1/2 phosphorylation, we found that both stimuli display distinct temporal courses. 8Br-cAMP (Fig 1B) and EGF (Fig 1C) induced maximal ERK1/2 phosphorylation at 15 and 5 min, respectively, followed by a fast decay up to the basal level in the EGF-treated cells. Instead, 8Br-cAMP sustained prolonged ERK1/2 activation, which was still evident at one hour of stimulation. In both conditions, ERK1/2 phosphorylation was almost completely inhibited by MEK1/2 inhibitor U0126 (Fig. 1B and C).

As shown previously [28], ERK1/2 phosphorylation by human gonadotropin (hCG) or 8Br-cAMP was inhibited when the cells were treated with H89, a PKA inhibitor (data not shown).

### Localization of pERK1/2 after stimulation by cAMP or EGF

**Figure 2:**
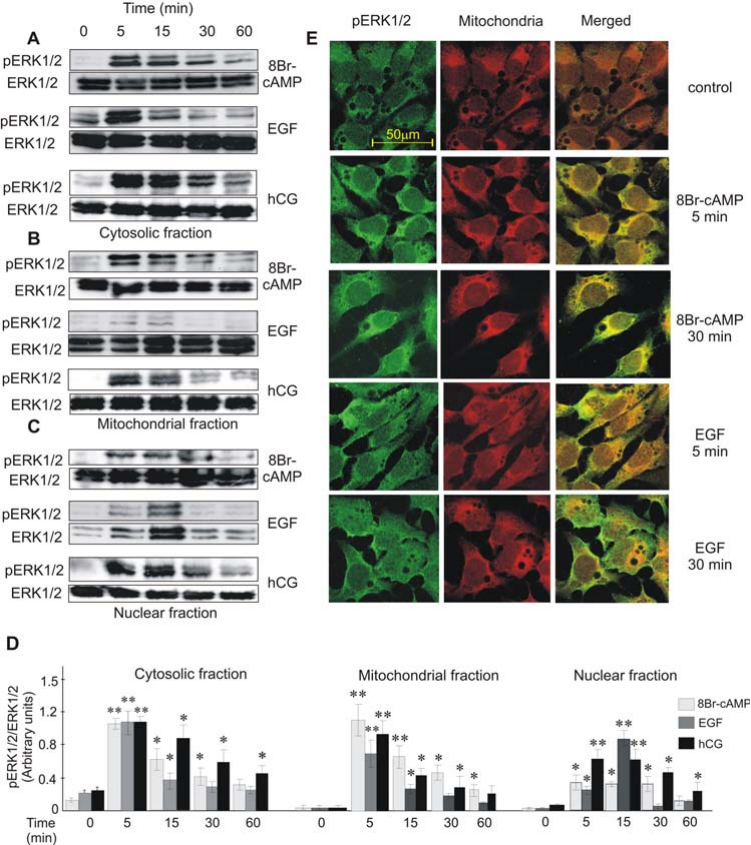
Comparison of subcellular distribution of hormone- or EGF-stimulated pERK1/2 activation. MA-10 cells were incubated with or without 0.5 mM 8Br-cAMP (upper panels of A, B and C), 10 ng/ml of EGF (middle panels of A, B and C) or 20 ng/ml of hCG (lower panels of A, B and C) for the indicated times. Next, subcellular fractions were obtained and 40 µg of the total protein of each fraction were subjected to SDS-PAGE and Western blot to detect pERK1/2 (indicated with pERK1/2 in A, B, and C). After stripping, total ERK1/2 (indicated with ERK1/2 in A, B, and C) was detected in the same membrane. The Western blots show the results of a representative experiment performed three times. D shows the quantification, performed as described in Figure 1. **, p<0.01, *, p<0.05 vs. time 0. (E) Immunofluorescent staining for pERK1/2 (green) and mitochondria (red) in MA-10 cells after treatment with or without 0.5 mM of 8Br-cAMP or 10 ng/ml of EGF for the indicated times. Merged images are shown in the right panel.

The immunoblots showed that after stimulation with 8Br-cAMP, phospho-ERK1/2 (pERK1/2) was located in the cytosol and mitochondria and, to a much lesser extent, in the nuclear fraction (Fig. 2, upper panels of A, B and C). In both the mitochondria and cytosol, an early peak of ERK1/2 phosphorylation was followed by a slow progressive decrease of the signal during the first hour of 8Br-cAMP action. On the contrary, after EGF stimulation, pERK1/2 is mainly localized in the cytosol and nucleus (Fig. 2, middle panels of A, B, and C). In mitochondria, activation peaked early (5 minutes) and then decayed. hCG stimulation led to ERK1/2 activation with a similar profile to that induced by 8Br-cAMP (Fig. 2, lower panels of A, B, and C). This later result validates the use of 8Br-cAMP to replace the hormone.

Confocal microscopy corroborated two distinct chronologies for mitochondrial ERK1/2 activation by 8Br-cAMP and EGF (Fig. 2E). As in immunoblots, pERK1/2 colocalized with mitochondria after 8Br-cAMP action, an effect observable as early as five minutes (Fig. 2E) and still evident 30 minutes later (Fig. 2E). In contrast, after EGF supplementation colocalization of pERK1/2 and a mitochondrial marker was brief (5 minutes, Fig. 2E) and not evident at 30 minutes (Fig. 2E).

**Figure 3:**
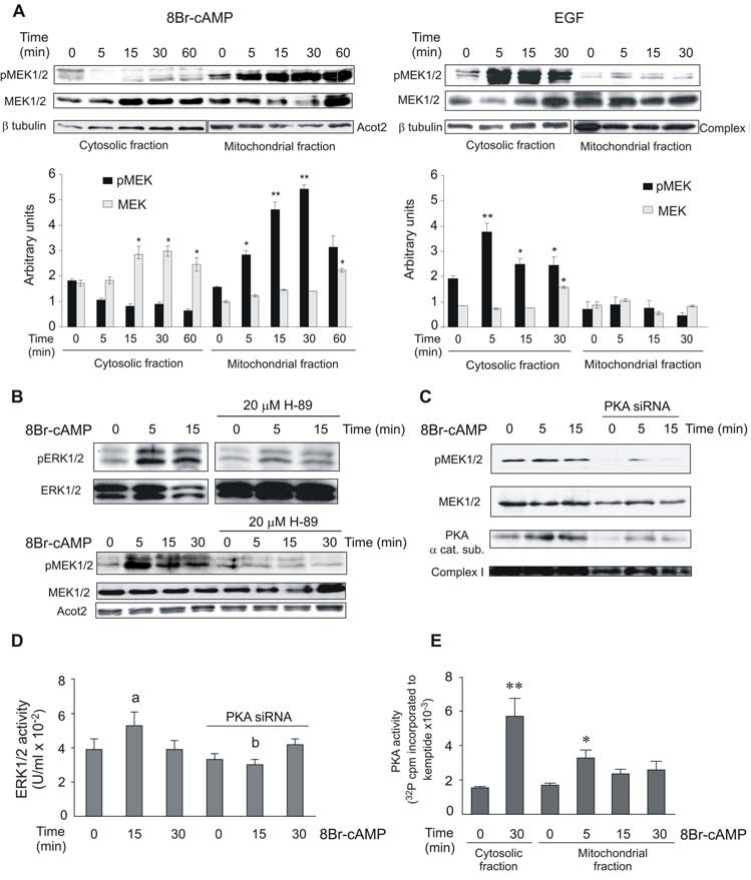
MEK1/2 activation in mitochondria and cytosol is entirely dependent on stimulus type and on PKA activity. (A) MA-10 cells were stimulated with 0.5 mM 8Br-cAMP or 10 ng/ml of EGF for the indicated times. Cytosolic and mitochondrial pMEK1/2 contents were analyzed by Western blot. A mitochondrial acyl-CoA thioesterase (Acot2), the 39 kDa subunit of the NADH-cytochrome c reductase (complex I) and cytosolic β-tubulin detection were used as loading control. The immunoblots show a representative result of three independent experiments. Bars denote levels of pMEK1/2 (black bars) and total MEK1/2 (grey bars) relative to β-tubulin (cytosolic fractions), Acot2 (mitochondrial fractions- 8Br-cAMP treatments) or complex I (mitochondrial fractions – EGF treatments) in arbitrary units. Data are expressed as means±SD of three independent experiments. * p<0.05 vs. 0 min; **, p<0.01 vs. 0 min. (B) Mitochondrial pERK1/2 and pMEK1/2 contents were analyzed in mitochondria obtained from MA-10 cells stimulated with or without 0.5 mM 8Br-cAMP for varying times, in the presence or absence of 20 µM of H-89, an inhibitor of PKA activity. Acot2 detection was used as loading control in pMEK1/2 and total MEK1/2 western blots. The panel shows a representative immunoblot from three independent experiments. (C) MA-10 cells were transiently transfected with 100 nM siRNA against the α isoform of the PKA catalytic subunit, using Lipofectamine 2000 reagent. After transfection, the cells were incubated with or without 0.5 mM of 8Br-cAMP for varying times and the contents of mitochondrial pMEK1/2, total MEK1/2, α isoform of the PKA catalytic subunit and the 39 kDa subunit of the NADH-cytochrome c reductase (complex I) were analyzed by western blot. This is a representative experiment from three separate experiments. (D) MA-10 cells were transiently transfected as described in (C) and stimulated with 8Br-cAMP for 15 and 30 minutes. pERK1/2 activity was measured using a pERK1/2 (pThr185/pTyr187) ELISA kit (Sigma Chemical Company, St. Louis, MO, USA), following the manufacturer's instructions. Bars represent the pERK1/2 activity as means±SD (n = 3). a, p<0.05, mock-transfected and 8Br-cAMP-treated cells (15 min) vs. mock-transfected and non-8Br-cAMP-treated cells (0 min); b, p<0.05, PKA catalytic subunit siRNA–transfected and 8Br-cAMP-treated (15 min) cells vs. mock-transfected and 8Br-cAMP-treated cells (15 min). (E) PKA activity in cytosolic and mitochondrial fractions isolated from MA-10 cells incubated with 0.5 mM 8Br-cAMP for varying times. Bars represent radioactivity incorporated into the kemptide-specific PKA synthetic substrate. The PKA assay was performed as previously described [61]. Data are expressed as means±SD (n = 3). **, p<0.01, * p<0.05 vs. 0 min.

We found two different pools of MEK1/2 and phospho-MEK1/2 (pMEK1/2) constitutively present in the cytosol and mitochondria of MA-10 cells (Fig. 3). Interestingly, MEK1/2 responded differently to stimulation depending on distribution (Fig. 3A). 8Br-cAMP clearly induced prolonged MEK1/2 phosphorylation in mitochondria, but had a less significant effect on the cytosolic kinases. Conversely, EGF induced a sustained and robust MEK1/2 activation in the cytosol but only a modest phosphorylation in mitochondria. Interestingly, although both EGF and 8Br-cAMP increased total cytosolic MEK1/2, only EGF promoted its phosphorylation in this subcellular fraction. Due to the fact that total MEK1/2 changed during the treatments, in the experiments depicted in Fig. 3, we also evaluated the contents of an acyl-CoA thioesterase (Acot2), 39 kDa subunit of the NADH-cytochrome c reductase (complex I) and β-tubulin in the corresponding blots. In cells stimulated with 8Br-cAMP, Acot2 was used as mitochondrial loading control [41]. In cells treated with EGF, Acot2 detection was replaced by the 39 kDa subunit of the NADH-cytochrome c reductase (complex I) since the content of the thioesterase changes with EGF treatment (unpublished observation). In both treatments, β-tubulin detection was used as cytosolic loading control. These loading controls indicated that the changes in total MEK1/2 signal are really due to a biological variation and not to different amount of proteins in each lane (15, 30 and 60 minutes of cAMP treatment in the cytosolic fraction and 60 minutes in the mitochondrial fraction; 30 minutes of EGF treatment in the cytosolic fraction). Therefore, we expressed pMEK1/2 relative to the loading control levels instead of relative to total MEK1/2 levels.

The increase of mitochondrial pERK1/2 and pMEK1/2 due to cAMP action was abolished by treatment of the cells with the PKA inhibitor H89 (Fig. 3B) and by PKA knockdown experiments (Fig. 3C and D). Accordingly, PKA activity in mitochondria showed a clear increase after 5 minutes of 8Br-cAMP action (Fig. 3E), in parallel with the appearance of the phosphorylated forms of MEK1/2 and ERK1/2 in the organelles.

The efficacy of the silence interference RNA (siRNA) treatment on the expression of the α isoform of the PKA catalytic subunit is demonstrated by the reduction in the cellular content of this isoform detected by western blot (Fig. 3C). As expected, the decrease in PKA protein levels results also in the reduction of cAMP-stimulated P4 production (P4 concentrations in the incubation media in ng/ml: control: 1.60±0.01; 8Br-cAMP 15 min: 3.7±0.6; siRNA+8Br-cAMP 15 min: 2.2±0.2, p<0.01). The reduction in progesterone production by PKA knockdown is less extensive than with the use of H89 (data not shown) since, as described above, the efficiency of the transfection is not 100% as it is the uptake of H89 by the cells.

### MEK phosphorylation *via* PKA together with StAR and pERK1/2 increase *ex vivo* cholesterol transport and mitochondrial synthesis of progesterone

**Figure 4:**
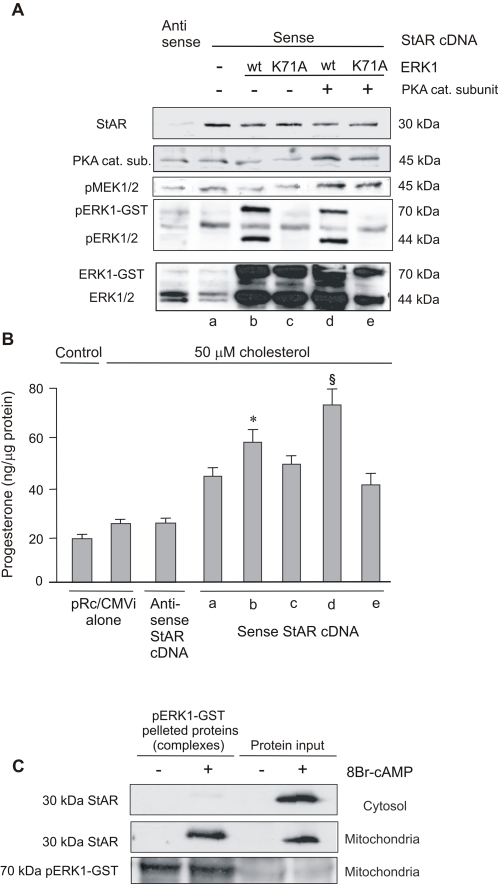
PKA and ERK1/2 are strictly required to achieve maximal progesterone production by isolated mitochondria. MA-10 cells were transiently transfected by electroporation with an empty vector or with a vector containing StAR cDNA (sense or antisense orientations). Mitochondria were incubated in the presence of cholesterol as substrate (a) in the presence of wild type ERK1-GST protein alone (b) or together with PKA catalytic subunit (d). The mutated inactive form of ERK1-GST (K71A) was also used (c and e). After the indicated incubations, mitochondria were pelleted and subjected to SDS-PAGE and Western blot (A). Specific antibodies that recognize StAR protein, the catalytic subunit of PKA, pMEK1/2, pERK1/2 and total ERK1/2 were used. The panel shows representative Western blots of three independently performed experiments. P4 production is shown in (B). Data are expressed as means±SD (n = 3). * p<0.05 bar b vs. bar a and § p<0.05 bar d vs. bar b. (C) MA-10 cells were treated with or without 0.5 mM of 8Br-cAMP for 3 hours; cytosolic and mitochondrial subcellular fractions were obtained and incubated in the presence or absence of human pERK1-GST bound to agarose beads. Input and pelleted proteins bound to pERK1-GST (complexes) were analyzed by SDS-PAGE and Western blot. The immunoblots show the bands corresponding to StAR and pERK1/2, as loading control. Data are representative of three independently performed experiments.

To test the direct effect of active ERK1/2 on cholesterol import and progesterone synthesis, we performed a cell-free assay [22], determining progesterone production by isolated mitochondria. To enrich non-stimulated mitochondria with StAR, we transfected StAR cDNA to MA-10 cells. Immunoblot showed that StAR expression was efficiently increased and processed in mitochondria (Fig. 4A). Transfection of full-length StAR cDNA in the sense orientation produced a small increase of P4 by mitochondria, as compared to the transfection with the empty vector or the antisense sequence (Fig. 4B, bar a vs. antisense and pRc/CMVi alone). After supplementation of isolated mitochondria with recombinant ERK1, the P4 yield increased (Fig. 4B, bar b vs. a). Addition of an inactive ERK1 mutant (K71A) did not affect steroid production (Fig. 4B, bar c vs. a). K71A lacks the capacity of autophosphorylation and has a reduced phosphotransferase activity [42]. Production of P4 increased more when the isolated mitochondria were supplemented with the recombinant PKA catalytic subunit and wild-type ERK1 than when they were supplemented with wild-type ERK1 alone (Fig. 4B, bar d vs. b). Again, this effect was lost when ERK1 was replaced by its K71A inactive form (Fig. 4B, bar e vs. d). Supplementation with wild-type ERK1, K71A ERK1, with or without the PKA catalytic subunit, did not affect StAR mitochondrial content (Fig. 4A). Also shown in Figure 4A is the mitochondrial content of PKA, pMEK1/2, pERK1/2 and total ERK1/2 after the corresponding incubation. It is worth to mention that in the mitochondrial samples incubated with ERK1-GST (Fig. 4A, lanes b, c, d and e) there is a strong signal of the 44 kDa ERK1, probably due to a cleavage of the fusion protein by mitochondrial proteases rendering the free protein [43]. The use of protease inhibitors is not indicated in this kind of experiment since mitochondrial steroidogenesis is dependent on StAR processing by these proteases [10], [44]. As already demonstrated in the *in vivo* experiments shown in Figure 3, the addition of PKA increased mitochondrial pMEK1/2 *in vitro* (Fig. 4A). These results support the possible formation of a large mitochondrial multi-kinase complex which leads to activation in steroid production.

### ERK1 interacts with StAR in mitochondria

Initially, we examined StAR structure to identify consensus sequences that would allow docking to ERK1/2. A typical docking site known as the D domain (**K**T**K**LTWL**LSI)** was found between amino acids 235 and 244. This site is conserved among ERK1/2 upstream kinases, MAPK phosphatases and ERKs substrates [45]. This finding led us to test protein-protein interactions in MA-10 subcellular fractions. To work with mitochondria enriched in StAR, we isolated this subcellular fraction from cells that had been stimulated for 3 hours with 8Br-cAMP. After this stimulation, the 30-kDa isoform of StAR was detected in the cytosolic and mitochondrial fractions (Fig. 4C). Treatment of the subcellular fractions with pERK1-GST showed that StAR interacts with pERK1 only in the mitochondrial fraction (Fig. 4C).

### StAR is phosphorylated by ERK1/2 *in vitro* and this phosphorylation is increased by the presence of cholesterol

**Figure 5:**
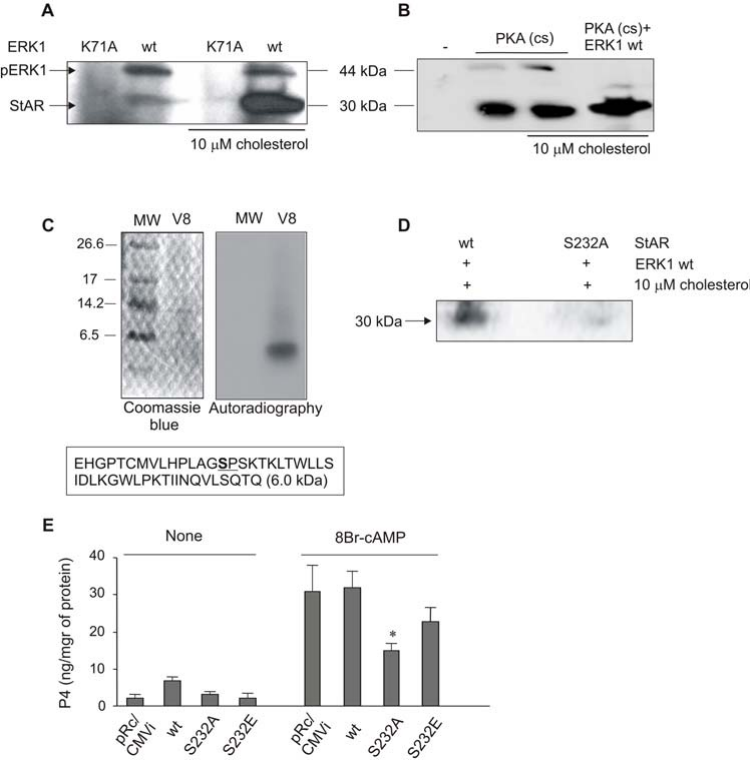
StAR is phosphorylated in Ser^232^ by ERK1 in a cholesterol-dependent manner. Thirty µg of recombinant purified 30 kDa wild-type StAR were incubated in the absence or in the presence of 10 µM of cholesterol (panels A and B) together with constitutively active His-tagged wild type ERK1 (wt) or the mutated inactive form of ERK1 (K71A) (panel A) or together with the catalytic subunit of PKA (cs) in the presence or absence of His-tagged wild type ERK1 (panel B). After the phosphorylation assays, phosphorylated protein levels were analyzed by SDS-PAGE and autoradiography. (C) StAR was phosphorylated by His-tagged wild type ERK1 in the presence of cholesterol as mentioned above. The phosphorylated product was subjected to limited digestion with endoprotease V8 as previously described [8]. Peptides were analyzed by gels as described by Shagger and Von Jagow [62] and by autoradiography. The box indicates the amino acid sequence of the 6 kDa peptide, where the Ser^232^ is underlined. (D) The wild type and a mutated form (S232A) of StAR were used in the *in vitro* phosphorylation assay described in (A). The phosphorylated products were analyzed by SDS-PAGE and autoradiography. The results in panels A, B, C, and D are representative of three independently performed experiments. (E) MA-10 cells were transfected by electroporation with an empty vector (pRc/CMVi) or with the vector containing the full length cDNAs of 37 kDa wild type StAR (wt), S232A StAR (S232A) or S232E StAR (S232E). The cells were stimulated with 0.5 mM of 8Br-cAMP for 30 minutes. P4 production was evaluated by determination of P4 concentrations in the incubation media by RIA. Data are expressed as means±SD (n = 3). *, p<0.05 S232A StAR vs. wt StAR.

To study the phosphorylation of StAR by ERK1/2, we performed an *in vitro* phosphorylation assay using the mature pure recombinant StAR protein (30 kDa) and two forms of ERK1 in the presence of [γ-^32^P]ATP. StAR was indeed phosphorylated by wild-type ERK1, but this phenomenon was not observed in the presence of the inactive mutant K71A of the kinase (Fig. 5A). Interestingly, phosphorylation by wild-type ERK1 seems to require cholesterol since the signal was notably increased in the presence of this lipid (Fig. 5A).

StAR phosphorylation by the PKA catalytic subunit was dependent neither on previous StAR phosphorylation by ERK1 nor on the presence of cholesterol (Fig. 5B). The combined action of PKA and wild-type ERK1 in the presence of cholesterol further increased the intensity of the radioactive signal, indicating a double phosphorylation of StAR by these two kinases (Fig. 5B).

### ERK1/2 phosphorylates StAR at Ser^232^

Next, we searched for the ERK1/2 phosphorylation site in StAR. Only two Ser-Pro motifs, targets for ERK1/2 phosphorylation, are detectable in the mature form of the murine StAR protein at Ser^232^ and Ser^277^. According to the database Expasy Prosite (http://expasy.org/prosite/), Ser^232^ has a 90% probability of being phosphorylated, whereas the probability for Ser^277^ is only 5%. Moreover, Ser^277^ is relatively less conserved than Ser^232^ among species (data not shown). Ser^232^ (PLAGS^232^PS) is adjacent to the docking D domain (−2). To demonstrate that Ser^232^ is the phosphorylation site of StAR by ERK1/2, we analyzed both the proteolytic products and the phosphorylation of a mutated form of StAR protein.

The 30 kDa phospho-StAR has been identified as the substrate of the protease V8 [8]. This protease hydrolyzes peptidic unions involving the α-carboxylic group of glutamic acid residues. According to the primary sequence of StAR, this protease would produce a small peptide of approximately 6 kDa containing the Ser residue that corresponds to the Ser^232^ in the StAR protein sequence. We performed the *in vitro* phosphorylation assay described in Fig. 5A. The phosphorylation was followed by phospho-StAR proteolysis by V8; the resulting peptides were analyzed by electrophoresis and autoradiography. A unique radioactive band was revealed at 6 kDa, which encompasses the mass of the expected peptide (Fig. 5C). Even when Ser^232^ is the only residue that could appear phosphorylated in this fragment, the confirmation that Ser^232^ is the target of ERK1/2 phosphorylation came from the experiments using S232A, a mutated form of StAR, in which Ser^232^ was changed to Ala, a non-phosphorylable amino acid. This mutated form of StAR was used in the *in vitro* phosphorylation assay using active ERK1 as kinase. The mutation impeded the phosphorylation of StAR by active ERK1, confirming that this residue is indeed the target of the kinase (Fig. 5D).

### StAR activity *in vivo* is reduced by mutation of Ser^232^

To study the role of Ser^232^ in StAR function, we transiently transfected MA-10 cells with two different StAR mutants. Progesterone production was enhanced by stimulation with 8Br-cAMP for 30 min; that enhanced production was partially blocked when the cells were transfected with the non-phosphorylable mutant of StAR (S232A) (Fig. 5E). Instead, the steroidogenic capacity was partially affected by the replacement of Ser with Glu (S232E) (Fig. 5E). This is probably due to the fact that Glu is a negatively charged amino acid that mimics the negative charge of the phosphate group present in the phospho-Ser.

## Discussion

In this study we have demonstrated that a) steroidogenesis in MA-10 Leydig cells depends on the specific temporal pattern of ERK1/2 activation in mitochondria; b) ERK1/2 phosphorylation is driven by mitochondrial PKA and constitutive MEK1/2 in the organelles; c) active ERK1/2 interacts with StAR and leads to its phosphorylation at Ser^232^; and d) double phosphorylation by PKA and ERK1/2 may favor StAR function.

We showed that PKA activates the constitutive mitochondrial MEK1/2 pool, a finding that accords with previous reports [28], [30], [31]. Interestingly, constitutive mitochondrial PKA-phosphorylated MEK1/2 preferentially activates a non-phosphorylated mitochondrial pool of ERK1/2 when the three kinases interact at the outer mitochondrial membrane, a crucial site of the steroidogenesis. It is clear that, under the influence of a classic ERK1/2 activator such as EGF and in the absence of mitochondrial PKA activation, the cytosolic and not the mitochondrial MEK1/2 pool is activated. Consequently, the two stimuli, 8Br-cAMP and EGF, favor scenarios of pERK distribution –localization in the mitochondria and steroidogenesis or in the nucleus and cell proliferation, respectively - that are qualitatively and quantitatively rather different.

**Figure 6:**
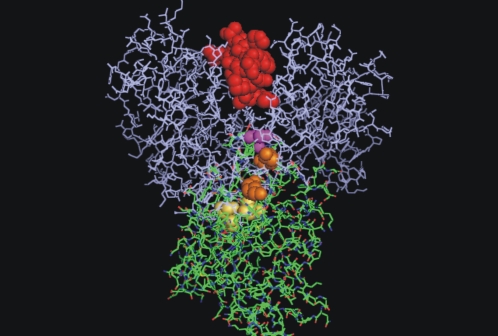
Predictive model of the molecular interaction between ERK1/2 and StAR. The reconstruction of molecular interaction of ERK2 and StAR was performed using PyMOL (DeLano Scientific, USA; www.delanoscientific.com). ERK2 (Protein Data Bank code 2GPH) is represented in blue and StAR (START domain in StartD4 from *Mus musculus*) in green. In this model, StAR is located in the docking groove of ERK2. The active center of ERK2 is in dark red. The CD domain of ERK2, represented in yellow, includes Asp^316^ and Asp^319^ in contact with the D domain of StAR. Lys^174^ and Lys^176^, corresponding to Lys^235^ and Lys^237^ of StAR sequence in *Mus musculus* are represented in orange, and Ser^171^, corresponding to Ser^232^ of murine StAR, in dark pink.

It was confirmed here that pERK1/2 in mitochondria have a functional interaction with StAR, MEK1/2 and PKA, thus forming a mitochondrial multi-complex. On the basis of crystallographic analysis, acidic and hydrophobic patches in the ERK1/2 structure (the CD domain) were described [46]. For instance, in the model of interaction between a peptide that belongs to MKP3 (MAP kinase phosphatase 3) and ERK2 [45], Asp^316^ and Asp^319^ of the ERK2 CD domain were observed to interact with Arg^20^ and Arg^21^ of the representative MKP3 peptide (R^20^R^21^GSNVALML, the D domain), an interaction putatively attributed in the present case to Lys^235^ and Lys^237^ of StAR (K^235^TK^237^LTWLLSI). Based on a computerized model of the ERK2-StAR complex (Fig. 6), we found a possible interaction between the ε-amine group of Lys^235^ in StAR structure and the carboxylic group of Asp^319 ^in ERK2 structure, separated by 11 Å. As in MKP3, other interactions of the hydrophobic motifs, underlined in the partial sequences, are expected to stabilize the StAR binding to the ERK docking groove [45].

We also confirmed that ERK1/2-StAR binding conducts to StAR phosphorylation at Ser^232^. This residue integrates the classic SP motif for phosphorylation of substrates by ERK1/2 [47]. In StAR, the SP motif is adjacent to the binding domain to ERK1/2, considerably augmenting the phosphorylating efficiency of the kinase [48]. This case is similar to that of the classical ERK1/2 substrate Elk-1 [49], although the SP position is inverted in respect to the docking domain. In addition, SP motifs susceptible to phosphorylation by ERK1/2 have a Pro residue in position −1 or −2, whereas StAR has a conserved Pro at position −3. However, as in other substrates, Pro is followed by Leu and there are no acidic residues in the motif [47].

StAR phosphorylation by ERK1/2 is not dependent on previous StAR phosphorylation by PKA but requires the presence of cholesterol. Some have suggested that StAR is a molten globule that changes its carboxyl-terminal helix when cholesterol approaches the hydrophobic surface [50], [51]. Cholesterol has been shown to act as an allosteric modulator, facilitating further binding of StAR to ligand [51]. In this case, the displacement of the C termini of StAR by cholesterol may increase the exposure of its docking domain to the ERK groove. The docking motif containing Lys^235^ in the StAR sequence and Asp^319^ in the ERK sequence constitute the closest interaction, separated by a short distance, approximately 11 Å (Fig. 6). This distance is not enough; then, the conformational changes that cholesterol and the disorganization of the carboxi terminal may provoke would contribute to a closer localization between Ser^232^ and the catalytic site of ERK, producing a more efficient phosphorylation. It is known that after the approximation of the docking site, there is a conformational change in the kinase that provokes the approximation of the phosphorylable site to the catalytic center [52].

Regarding the form of StAR protein that was subjected to *in vitro* phosphorylation, several reasons explain the election of the 30 kDa protein. First, the bibliography is controversial, since there is not a full explanation of StAR protein mitochondrial import, maturation/activation and function. In this regard, there is not conclusive evidence to assign the activity to either of the two isoforms, 30 or 37 kDa. Second, in our pull down experiments, described above, ERK1 specifically co-precipitates with the 30 kDa mitochondrial form of StAR protein, indicating that interaction between ERK and this isoform is probably after StAR protein cleavage. Thus, these facts support the concept that the 30 kDa form of StAR could be the substrate of ERK1/2. Nevertheless, the transfection experiments, performed with a mutated form of the full-length form of StAR (37 kDa), indicate that being the 37 or the 30 kDa forms the substrate for ERK1/2, the phosphorylation of StAR at Ser^232^ is necessary for its steroidogenic activity.

The presence of a multi-protein complex in the outer mitochondrial membrane has functional repercussions for steroidogenesis. In this complex, phosphorylation of StAR by ERK1/2 is a key process for cholesterol transport. It is known that StAR works together with PBR which in turn interacts with VDAC. However, little is known about how StAR interacts with these proteins. In previous unpublished observations we detected that StAR could interact with VDAC. It will be interesting to further analyze the role of Ser^232^-StAR phosphorylation in the assembly of this multiprotein complex and in its function. The additional negative charges in the StAR (30 kDa) molecule due to phosphorylation at Ser^232^ may contribute to the retention of the mature form of 30 kDa StAR in the outer mitochondrial membrane and to the formation of the multiprotein complex.

## Materials and Methods

### Antibodies and reagents

Purified hCG was a kindly provided by Dr. Parlow (National Hormone and Pituitary Program, National Institute of Diabetes & Digestive & Kidney Diseases (NIDDK, NIH, Bethesda, MD, USA). Anti-phospho-ERK1/2, anti-phospho-MEK1/2, anti-total MEK1/2 antibodies and U0126 were obtained from Cell Signaling (Beverly, MA, USA); anti-total ERK1/2 and anti-β-tubulin were purchased from Upstate Biotechnology (Lake Placid, NY, USA). Anti- 39 kDa subunit of the NADH-cytochorme c reductase was obtained from Molecular Probes, Inc. (Eugene, OR, USA). Anti-StAR was generously provided by Dr. Douglas Stocco (Texas Tech University Health Sciences Center, Lubbock, TX, USA). The antibody that recognizes the PKA α catalytic subunit (Santa Cruz Biotechnology, CA, USA) was a generous gift from Dr. Tellez Iñón (INGEBI, Buenos Aires, Argentina). The antibody against Acot2 was generated at our laboratory [53]. Wild-type and a catalytically inactive variant (K71A) of human ERK1 fused to GST were purchased from Stressgen (Ann Arbor, MI, USA). Wild-type ERK1-GST was activated *in vitro* in accordance with the manufacturer's instructions. Constitutively active His-tagged ERK1 and PD98059 were purchased from Calbiochem (San Diego, CA, USA). The PKA catalytic subunit was obtained from New England Biolabs (Beverly, MA, USA). Site-directed mutagenesis on StAR wild-type cDNA was performed on a pRc/CMVi construction by GenScript (GenScript Corporation, Piscataway, NJ, USA). The siRNA against the PKA α catalytic subunit was obtained from Santa Cruz Biotechnology (Santa Cruz Biotechnology, CA, USA). The 3×flag-CMV7-ERK2 plasmid containing the wild-type or a mutated (H230R) variant of ERK2 was kindly provided by Dr. Melanie H. Cobb (Department of Pharmacology, University of Texas Southwestern Medical Center, Dallas, TX, USA). All others reagents were commercial products of the highest grade available.

### Cell Culture

The MA-10 cell line is a clonal strain of mouse Leydig tumor cells that produces progesterone (P4) rather than testosterone as the major steroid [54]. MA-10 cells were generously provided by Dr. Mario Ascoli, University of Iowa, College of Medicine (Iowa City, IA, USA) and were handled as originally described [41], [54].

### Cell transfection and constructions

Cell transfections were performed by electroporation or using a cationic lipid reagent. MA-10 cells were transiently transfected by electroporation in accordance with already published procedures [41] with an empty vector or with a vector containing StAR cDNA (sense or antisense orientations). The incorporation of the siRNA of the α isoform of the catalytic subunit of PKA into the cells was performed using Lipofectamine 2000 reagent (Invitrogen, Carlsbad, CA, USA), according to previously used procedures [37].

Full length StAR cDNA (Gen Bank accession n° BC082283) was obtained by PCR from MA-10 cells as described in Maloberti *et al.* [37]. Primers were designed according to the published sequence of mouse StAR. The forward (5′-GGACCTTGAAAGGCTCAGGAAGAACAACCC-3′) and the reverse (5′-GGATTAGTAGGGAAGTCGGCACAATGATGG-3′) primers were used to amplify a 1440-bp fragment. The sequence was subcloned in the eukaryotic expression plasmid, pRc/CMVi, a gift from Ingo Leibiger from Karolinska Institut, Stockholm, Sweden. The ligation provided both sense and antisense orientations that were used for transformation of XL-1 *E. coli*. Then, several clones were screened and sense and antisense plasmids were obtained by midipreparations with the Wizard® system (Promega, Madison, WI, USA); afterwards used for transfections of MA-10 cells.

Recombinant 30 kDA StAR protein was obtained as described before [15]. Briefly, StAR cDNA for the mature form (30 kDa) was obtained by PCR from MA-10 cells with the following primers: the forward 5′-GGATCCGCAGGGTGGATGGGTCAA-3′ and the reverse 5′-GGATTAGTAGGGAAGTCGGCACAATGATGG-3′ that amplify a 1200-pb fragment. Then, the StAR cDNA was cloned in a pGEX-4T-3 plasmid (Promega, Madison, WI, USA). The construction was used to transform BL-21 *E. coli*, the recombinant protein GST-StAR was purified by glutathione affinity chromatography (GST Purification Module, Amersham Biosciences, Sweden) and subjected to thrombin proteolytic cleavage. Then, *in vitro* phosphorylation experiments were performed with 30 kDa StAR. The same protocol was followed to obtain a mutated form of the StAR protein (S232A) using the site-directed mutated StAR cDNA.

### Subcellular fractionation

Subcellular fractionation was performed as described previously; nuclear, cytosolic and mitochondrial fractions from MA-10 cells were isolated by differential centrifugation [22], [34], [55]. A detailed description of the procedure is the following. After the corresponding treatments, culture media was removed, MA-10 cells were washed with PBS supplemented with protease and phosphatase inhibitors and collected by centrifugation at 800×*g* for 10 minutes. MA-10 cells were resuspended in MSHE buffer (219 mM D-mannitol, 70 mM sucrose, 0.02% EGTA, 0,1% BSA, 1.8 mM Hepes pH 7.4) and subjected to mechanical disruption with 60 strokes with an insulin syringe. Then, nuclear fraction was obtained by centrifugation at 5000×*g* for 10 minutes. The supernatant is centrifuged at 15000×*g* for 30 minutes. Then, the pellet corresponding to the mitochondrial fraction was washed once and resuspended in MSHE buffer and supernatant corresponds to cytosolic fraction. Fractions were subjected to enzymatic analysis to assess their purity (according to [34]). The purity of each fraction was at least 90%, value similar to previous publications [56].

### Cell-free assay for mitochondrial steroidogenesis

After transfections, MA-10 cells were subjected to subcellular fractionation in order to obtain mitochondria. The organelles were resuspended in 10 mM of malate, 1 mM of Mg_2_Cl, 50 µM of ATP, 40 mM of β-Glicerophosphate, 1 mM of DTT. Stimulation of mitochondrial steroidogenesis was performed according to published procedures [22], [55]. Mitochondria were incubated in the absence or presence of 50 µM of cholesterol as substrate for 20 minutes at 30°C. Cholesterol supplementation was performed in the presence or absence of 1 µg of wild- type ERK1-GST protein alone or together with 1 IU of PKA catalytic subunit. The mutated inactive form of ERK1 (K71A) was also used. The K71A mutant of ERK1 was described by Charest *et al.* [42]. The change in one aminoacidic residue abolishes the autophosphorylation activity of the kinase and the myelin basic protein phosphotransferase activity. While this mutated form is able to be phosphorylated by MEK1/2 to approximately 80% of the level achieved with its non-mutated form, the result is a partial activation of the myelin basic protein phosphotransferase activity (20%). After the indicated incubations, P4 production in the incubation media was determined by radioimmunoassay [41] while mitochondrial protein content was analyzed by SDS-PAGE and Western blot.

### Pull-down assay

To study interaction between StAR protein and pERK, pull-down assays were carried out as indicated in Hirakawa and Ascoli [28]. Briefly, cytosolic or mitochondrial fractions (500 µg or 300 µg, respectively) were incubated in the presence of human recombinant ERK1-GST coupled to agarose beads, previously activated as described by the manufacturer. The incubations were performed in pull down buffer; 50 mM Tris pH 7.4, 150 mM NaCl, 1 mM EDTA, 1 mM EGTA, 10% glycerol, 0.5% Nonidet P-40, 1 mM MgCl_2_ supplemented with protease and phosphatases inhibitors overnight at 4°C. Afterwards, proteins bound to ERK1-GST contained in the agarose-protein pellet (complexes) were washed three times with the same buffer, boiled for 5 minutes, subjected to SDS-PAGE and immunoblot analysis.

### *In vitro* phosphorylation of recombinant StAR

Phosphorylation assays of wild-type or S232A StAR were performed with wild-type His-tagged ERK1 or the catalytically inactive form of ERK1-GST (K71A) and PKA catalytic subunit. The assays were performed with 10 µCi of [γ-^32^P]ATP for 30 minutes at 30°C as described previously [49], [57].

### SDS-PAGE and immunoblot assay

Proteins were separated by SDS-PAGE and electrotransferred to poly-(vinylidine difluoride) (PDVF) membranes as previously described [58]. Immunodetection was performed using the specific antibodies described in the figure legends. Antibodies recognizing pERK and total ERK were used sequentially in the same membrane after treatment of the blots with stripping buffer (62.5 mM Tris, pH 6.8, 100 mM 2-mercaptoethanol and 2% SDS) for 30 minutes at 60°C. The same statement is valid for pMEK and total MEK recognition. In the western blots evaluating pMEK1/2 content, total MEK1/2 and other proteins were used as loading control: a mitochondrial acyl-CoA thioesterase for 8Br-cAMP treatment as previously described [41], the mitochondrial 39 kDa subunit of the NADH-cytochrome c reductase (complex I) for EGF treatment, and cytosolic β-tubulin in both cases. Proteins were visualized using horseradish peroxidase-coupled secondary antibodies followed by the ECL chemiluminescence detection system (Amersham Pharmacia Biotech, Piscataway, NJ, USA) and X-ray film exposure. For quantitative analysis, densitometry was performed using a Storm Phosphorimager scanner (Amersham Biosciences, Sweden) and band intensities were analyzed using ImageQuant 5.2 software.

### Immunofluorescence analysis

MA-10 cells were grown to approximately 80% confluence on poly-L-lysine glass coverslips. After treatments, cells were incubated for 45 minutes at 36.5°C with 300 nM Mitotracker Red® (Molecular Probes, Inc., Eugene, OR, USA), a specific mitochondrial dye. Then, cells were fixed with 4% paraformaldehyde in PBS for 10 minutes at room temperature and permeabilized with blocking solution (0.3% Triton X-100 and 1% BSA in PBS) for 60 minutes at room temperature. The detailed procedure was described previously [59]. Cells were incubated with anti-pERK1/2 cy2-conjugated antibody (1∶250) overnight at 4°C. After several washes with PBS, cells were incubated for 1h at room temperature with a goat anti-rabbit antibody (1:400). Coverslips were mounted onto the slides using Fluorsave antifade reagent (Calbiochem), followed by confocal analysis using a Nikon C1 microscope (IByME, UBA, Argentina).

### Statistical analysis

Data were analyzed by ANOVA followed by the Dunnet test.
